# Effect of Vitamin E Supplementation on Chronic Insomnia Disorder in Postmenopausal Women: A Prospective, Double-Blinded Randomized Controlled Trial

**DOI:** 10.3390/nu15051187

**Published:** 2023-02-27

**Authors:** Wirun Thongchumnum, Sakda Arj-Ong Vallibhakara, Areepan Sophonsritsuk, Orawin Vallibhakara

**Affiliations:** 1 Reproductive Endocrinology and Infertility Unit, Department of Obstetrics and Gynaecology, Faculty of Medicine, Ramathibodi Hospital, Mahidol University, Bangkok 10400, Thailand; 2 Faculty of Medicine, BangkokThonburi University, Bangkok 10170, Thailand; 3 Child Safety Promotion and Injury Prevention Research Center, Faculty of Medicine, Ramathibodi Hospital, Mahidol University, Bangkok 10400, Thailand

**Keywords:** chronic insomnia disorder, postmenopausal women, vitamin E, The Pittsburgh Sleep Quality Index, PSQI, insomnia, sleep disturbance, supplement

## Abstract

Chronic insomnia disorder is one of the most common problems in postmenopausal women, exacerbated by underdiagnosis and improper treatment. This double-blinded, randomized, placebo-controlled trial was conducted to evaluate the potential of vitamin E to treat chronic insomnia as an alternative to sedative drugs and hormonal therapy. The study enrolled 160 postmenopausal women with chronic insomnia disorder, divided randomly into two groups. The vitamin E group received 400 units of mixed tocopherol daily, while the placebo group received an identical oral capsule. The primary outcome of this study was sleep quality assessed by the Pittsburgh Sleep Quality Index (PSQI), a self-evaluated and standardized questionnaire. The secondary outcome was the percentage of participants using sedative drugs. There were no significant differences in baseline characteristics between the study groups. However, the median PSQI score at baseline was slightly higher in the vitamin E group compared with the placebo (13 (6, 20) vs. 11 (6, 20); *p*-value 0.019). After one month of intervention, the PSQI score was significantly lower (indicating better sleep quality) in the vitamin E group compared with the placebo (6 (1, 18) vs. 9 (1, 19); *p*-value 0.012). Moreover, the improvement score was significantly higher in the vitamin E group compared with the placebo (5 (−6, 14) vs. 1 (−5,13); *p*-value < 0.001). In addition, there was a significant reduction in the percentage of patients using sedative drugs in the vitamin E group (15%; *p*-value 0.009), while this reduction was not statistically significant in the placebo group (7.5%; *p*-value 0.077). This study demonstrates vitamin E’s potential as an excellent alternative treatment for chronic insomnia disorder that improves sleep quality and reduces sedative drug use.

## 1. Introduction

Postmenopausal women are defined as women with amenorrhea for at least 12 consecutive months due to the cessation of ovarian function. The average age of natural menopause is marginally different across ethnicity and is 49.5 years old for Thai women [1]. Having an average life expectancy at birth of 80.5 years [2], an increased number of aging Thai women are experiencing menopausal symptoms such as vasomotor symptoms, vulvovaginal atrophy, and long-term consequences including dementia, increased risk of cardiovascular disease, and osteoporosis [3]. Another common health problem among them is insomnia, which is defined as difficulties initiating or maintaining sleep or early morning awakening associated with impaired daytime cognitive performance, fatigue, or mood disturbances [4]. The etiologies of insomnia are based on the “3Ps” model: predisposing, precipitation, and perpetuating factors [5]. With an age older than 45 years and being in the menopause transition period, women are more likely to have predisposing factors for insomnia compared with men [6]. The menopausal symptoms, such as hot flushes, night sweats, and nocturia, are the precipitating factors. The perpetuating factor becomes operative when insomnia transitions from acute to chronic.

Insomnia involves hyperarousal stages during sleep and wakefulness with increased arousal levels in the cognitive, emotional, and physiologic domains. This manifests as an elevated, whole-body metabolic rate during sleep and wakefulness, elevated cortisol and adrenocorticotropic hormone during the early sleep period, and reduced parasympathetic tone in heart rate variability. Moreover, there is evidence that insomniacs have increased power in fast electroencephalographic (EEG) frequencies during non-rapid eye movement (NREM) sleep and an increased frequency of microarousals during rapid eye movement (REM) sleep, leading them to perceive parts of REM sleep to be wakefulness [5].

The prevalence of insomnia among the general population is about 10%, with a female and increasing-age preponderance. This places menopausal women at high risk. Sleep complaints increased dramatically during the menopausal transition, with the prevalence increasing from 12 to 40% from late reproductive age (around the late 40s) into perimenopause (in the early 50s) [7]. Moreover, the prevalence of insomnia among menopausal women was twice that of reproductive-age women (26% versus 13%). The changes in the levels of various sex hormones—including decreases in estradiol level and increases in the follicle-stimulating hormone, progesterone, and testosterone across the menopausal transition—are associated with perceived poor sleep quality, sleep fragmentation, and increased awakenings. Medical conditions that increase during midlife, including obesity, cancer, gastroesophageal reflux, urinary incontinence, nocturnal micturition, thyroid dysfunction, chronic pain syndromes, and fibromyalgia, add to the impact of age on sleep. 

The available modalities to evaluate sleep disturbance are subjective insomnia rating scales or questionnaires and objective devices, such as wrist actigraphy and polysomnography [5]. However, the mixed results yielded by objective sleep monitoring and polysomnography (PSG) have inconsistently evidenced improvement or worsening of sleep patterns in the menopausal transition [8]. Young T et al.’s report of sleep patterns from PSG among premenopausal, perimenopausal, and postmenopausal women showed no significant difference across all groups [9]. In contrast, Xu M et al. revealed a worsening sleep pattern in menopausal women, defined by longer total wake time, and lower sleep efficiency compared to premenopausal women [10]. Due to these inconsistencies, PSG is not recommended for the evaluation of insomnia, despite its merits as an evaluation tool for sleep apnea or parasomnias. The effect of insomnia on health outcomes has been established. Not only does insomnia significantly increase the risk for many chronic diseases including arterial hypertension, myocardial infarction, heart failure, type 2 diabetes, cognitive impairment, neurodegenerative disease, major depression, sick leave, and accidents, both at work and in motor vehicles [4], but it also has the effect of increasing mortality. A recent meta-analysis by Ge L et al. aimed to assess the association between insomnia disorder and mortality risk. Insomnia is linked to an increased risk of all-cause mortality (hazard ratio (HR) 1.23, 95% CI 1.07–1.42, *p* = 0.003) and cardiovascular disease mortality (HR 1.48, 95% CI 1.07–1.42, *p* = 0.003) but not cancer-related mortality [11].

Chronic insomnia disorder is diagnosed based on the American Academy of Sleep Medicine’s International Classification of Sleep Disorders, Third Edition, which defines diagnostic criteria as a disturbance of nocturnal sleep at least three times a week for at least three months. Disturbances include difficulty in the initiation or maintaining of sleep or waking up earlier than desired and related daytime impairment such as fatigue, sleepiness, mood disturbance, and impaired cognitive and work performance [12]. Chronic insomnia is associated with a greater risk of mortality, morbidity, and accidents compared to acute insomnia [13,14]. The incidence of chronic insomnia in community-dwelling adults from cohort studies in the U.S., U.K., and Taiwan was 2–7% per year [15]. Incidence was much higher among postmenopausal women and more prevalent in the late than early stage of menopause, as reported by The Study of Women Across the Nation (SWAN), which found around one-third of women reporting chronic insomnia by the end of their menopausal transition [16]. In line with that cross-sectional, Punyahotra S. et al. revealed a 40% prevalence of insomnia among mid-aged Thai women [17]. 

Insomnia symptoms are often neglected by both patients and doctors, especially in gynecological practice. As a result, patients are not receiving effective treatment, which leads to persistent insomnia. Not only does chronic insomnia produce numerous ill-health outcomes, but it also affects the quality of life of menopausal women. Yazdi and colleagues evaluated the effect of insomnia on the quality of life among menopausal women through the Menopause-Specific Quality of Life (MENQOL) Questionnaire, a 29-item questionnaire that assesses the effects of menopausal symptoms on four categories of quality of life: vasomotor, psychosocial, physical, and sexual. The study showed that postmenopausal women with chronic insomnia disorder had significantly worse quality of life across all domains of their score [18]. 

The roles of oxidative stress and chronic sleep deprivation have been evaluated in many studies. This complex relationship works both ways; while oxidative stress may cause sleep disturbance by breaking the sleep–wake cycles, poor sleep quality also increases oxidative stress and lowers antioxidant levels [19]. The central nervous system (CNS), which contains high levels of polyunsaturated fatty acids (PUFAs) and lipids, is a vulnerable and well-known target of reactive oxygen species (ROS) [20]. Furthermore, the association between oxidative stress and various neurodegenerative diseases, such as Alzheimer’s disease, Amyotrophic lateral sclerosis, Friedreich’s ataxia, Huntington’s disease, Multiple sclerosis, and Parkinson’s diseases, was also well described [21]. Therefore, El-Helaly M. and a colleague investigated participants’ exposure to shallow frequency electromagnetic fields (ELF-EMF) and the relationship between oxidative stress and sleep quality in the clinical setting, which suggested that ELF-EMF exposure generates ROS, reflected by the high level of serum malondialdehyde level, and low melatonin, a scavenger of ROS. Undoubtedly, participants in the exposure group had higher ROS, lower levels of melatonin, and a more frequent prevalence of sleep insufficiency when compared to controls [22]. The study by Gulec and colleagues showed that the serum level of malondialdehyde (oxidative stress marker) in patients with primary insomnia was significantly higher than in controls. In the same way, insomnia was detrimental to antioxidant levels, where glutathione peroxidase was much lower in the poor sleep group [23]. Feng and colleagues discovered the same result in animal model research [24]. 

Vitamin E, which can be found in beans, vegetable oil, or supplement drugs, is an antioxidant that acts to reduce the destruction of cell membranes in the body by eradicating free radicals and reducing inflammation. Two main isoforms of vitamin E are tocopherol and tocotrienol. Each isoform is divided into four distinct analogs, including alpha (α), beta (β), gamma (γ), and delta (δ), which all have antioxidating properties and act as free radical scavengers [25]. Currently, the Recommended Dietary Allowance (RDA) is only given for alpha-tocopherol supplementation. The RDA for both male and female adults is 33.1 IU per day, and the maximum dose is 1000 IU per day [26]. The use of vitamin E is now more prevalent among postmenopausal women as a dietary supplement or an anti-aging agent. Large numbers of studies have shown that vitamin E may help with hot flushes caused by estrogen deficiency compared to placebo [27] or have a beneficial effect on bone formation and destruction in postmenopausal women with no effect on all-cause mortality [28,29,30,31]. Although there was no clinical trial performed to determine the potential of vitamin E for treating chronic insomnia disorder in humans, some studies found that vitamin E consumption significantly restored normal blood levels of glutathione peroxidase while suppressing malondialdehyde [32,33,34], which leads us to propose that a reduction in oxidative stress may be the mechanism by which vitamin E improves sleep quality.

There are several different approaches to treating chronic insomnia disorder including menopause hormone therapy, Cognitive Behavioral Therapy for Insomnia (CBT-I), and alternative herbal medicines such as Valerian and isoflavones, according to a 2016 publication by the North American Society of Menopause [12]. To build on their recommendations, the researchers conducted this study of the potential of vitamin E to treat chronic insomnia as an alternative to drugs with harmful side effects, especially hormone replacement therapy and sedatives.

## 2. Materials and Methods

### 2.1. Study Design

A double-blinded, randomized, placebo-controlled trial was conducted at the Department of Obstetrics and Gynaecology, Faculty of Medicine, Ramathibodi Hospital, Bangkok, Thailand, between November 2021 and May 2022. The study aimed to evaluate the effects of oral mixed tocopherol on postmenopausal chronic insomnia disorder. The results were measured by a subjective, self-evaluated questionnaire, the Pittsburgh Sleep Quality Index (PSQI), after one month of intervention. The PSQI was first developed in 1988 by Dr. Daniel J. Buysse and colleagues at the University of Pittsburgh. The questionnaire is intended to be a highly effective, standardized tool for assessing a patient’s sleep quality with a test reliability coefficient (Cronbach’s alpha) of 0.83, a sensitivity of 89.6%, and a specificity of 86.5% for sleep disorders [35]. The questionnaire registers a score from 0 (no sleep disturbance) to 3 (severe sleep disturbance) for each of the 7 components: subjective sleep quality, sleep latency, duration, habitual sleep efficiency, sleep disturbances, use of sleeping medication, and daytime dysfunction. Possible scores range from 0 to 21, and a value of more than 5 indicates poor sleep quality. The PSQI questionnaire has been translated into more than 56 languages, demonstrating its international recognition. Furthermore, the questionnaire was already translated and validated in Thai by Sitasuwan and colleagues in 2014 [36]. The primary outcome of this study was post-intervention sleep quality as assessed by the PSQI, a self-evaluated and standardized questionnaire. The secondary outcome was the reduction of sedative drugs used.

### 2.2. Participants

Postmenopausal women, defined as women who have had amenorrhea for a consecutive 12 months or who have undergone previous bilateral oophorectomy, who presented with chronic insomnia disorder meeting the diagnostic criteria of the American Academy of Sleep Medicine’s International Classification of Sleep Disorders Third Edition [12] and who had a PSQI score > 5 were included. Informed consent was provided by all participants. Women with hot flushes, with a contraindication to vitamin E intake (for example, taking an antiplatelet or anticoagulant or severe hepatic or renal disease), taking vitamin E or any other herbal medicine that may affect sleep, with an underlying psychiatric disease with prescribing medication, consuming caffeine more than two shots per day, working night shifts, undergoing cancer therapy, and with uncontrolled diabetes or hypertension were all excluded. 

### 2.3. Sample Size Calculation

The sample size calculation was based on the difference in sleep efficiency score in the exploratory study by Lichstein et al. [37] with the n4Studies application for randomized-controlled trials (version 1.4.1) [38]. Finally, with a power of 0.8, there were 72 participants in each study group. A total of 160 postmenopausal women were included in this study (accommodating for 10% data loss). 

### 2.4. Randomization, Blinding, and the Study Protocol

All participants were assigned using a computer-generated randomization sequence into each study group (1:1; blocks of four). The participants and investigators were blinded to the group allocation. The mixed tocopherol used for the intervention (Nat E^®^ (Mega Lifesciences Public Company Limited, Samutprakarn, Thailand), 400 units per tablet) contained 20% delta-tocopherol, 1% beta-tocopherol, 62% gamma-tocopherol, and 10% alpha-tocopherol. Participants in the vitamin E group received one tablet daily for one month.

In contrast, those in the placebo group received identical placebo capsules once daily for one month. Participants were encouraged to perform normal daily activities and to avoid other drugs besides the assigned intervention. However, participants could continue taking medication for underlying diseases or previous sedative drugs. 

### 2.5. Data Collection and Measurements

The participants’ baseline characteristics were collected at the time of enrollment, including age, body mass index (BMI), age at menopause, years since menopause, type of menopause, marital status, number of children, education, socioeconomic status, underlying disease, history of sedative drugs used, and caffeine consumption. In the first and third weeks, the investigators contacted all participants by phone or social media application to monitor drug compliance and side effects. This study defined good drug compliance as all participants taking more than 80% of the assigned drugs. The participants completed the PSQI questionnaire at the time of enrollment and at the end of one month of intervention.

### 2.6. Statistical Analysis

Baseline characteristics are reported as descriptive data. The normal distribution was tested for continuous variables with the Shapiro-Wilk normality test. When the data were normal or non-normally distributed, the mean (standard deviation, S.D.) or median (range) were used, respectively. The discrete variables were reported in counts (percentages). Statistical analysis was performed by STATA Version 15.0 (College Station, TX, USA). The student t-test was used to compare the continuous variables in parametric data. The Mann–Whitney U test was used for nonparametric data to compare continuous variables. The categorical data were tested by Pearson’s chi-squared test or Fisher exact test. A *p*-value of 0.05 defined statistical significance. Intention-to-treat analysis was used in this clinical trial.

### 2.7. Ethical Approval

Human Research Ethics Committee, Faculty of Medicine Ramathibodi Hospital, Mahidol University approved this study (MURA2021/819). In addition, the study protocol was submitted to the Thai Clinical Trials Registry; TCTR (www.thaiclinicaltrials.org access on 21 April 2022); clinical trial registration number: TCTR20220405002.

## 3. Results

### 3.1. Protocol Flow Diagram

**Figure 1:**
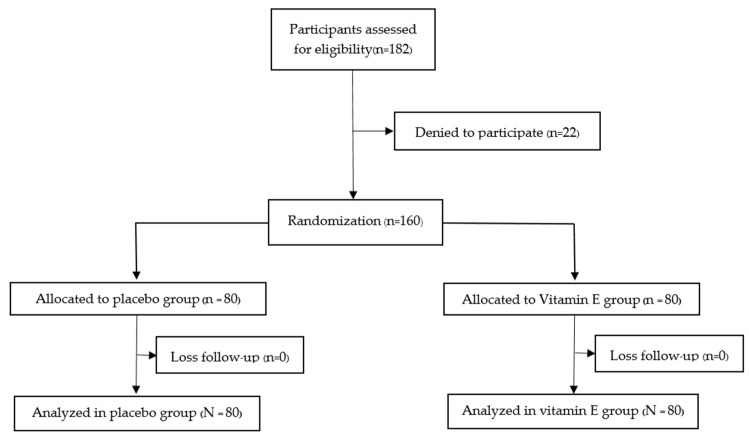
Protocol flow diagram of the study.

As shown in the protocol flow diagram (Figure 1), one hundred eighty-two postmenopausal women met the eligibility criteria, and twenty-two participants lost contact. A total of 160 participants were included in our study, and all participants were randomly and equally separated into the vitamin E and placebo groups. Fortunately, there were no losses to follow up in our study. The reason could be our design of a short study period of one month and the follow-up strategy, in which the authors contacted participants at least twice (in the first and third weeks) to monitor compliance and side effects. Furthermore, participants had the willingness to overcome chronic insomnia. As a result, all the participants cooperated well with the study’s design and reported no serious adverse effects.

### 3.2. Main Results

As shown in Table 1, baseline characteristics between the two study groups showed no statistical difference, including age, body mass index (BMI), age at menopause, years since menopause, type of menopause, marital status, number of children, education, socioeconomic status, underlying diseases, and caffeine consumption. There was a higher rate of sedative drug use in the vitamin E group compared to the placebo group, but there was no statistical significance. All of the prescribed sedative drugs in both study groups were benzodiazepines (either lorazepam or alprazolam). Most participants had well-controlled underlying diseases such as diabetes, dyslipidemia, and hypertension. No diseases could affect sleep, as described in our exclusion criteria. The primary outcome of this study was to evaluate the improvement in PSQI scores after one month of intervention. The pre-intervention PSQI scores of the two groups were marginally different (11 (6, 20) in the placebo group and 13 (6, 20) in the vitamin E group; *p*-value 0.019). This event was also observed in the previous study [36]. After the intervention, participants in the vitamin E group had significantly better sleep quality compared to the placebo group, with a score of 6 (1, 18) vs. 9 (1, 19) (*p*-value 0.012).

**Figure 2:**
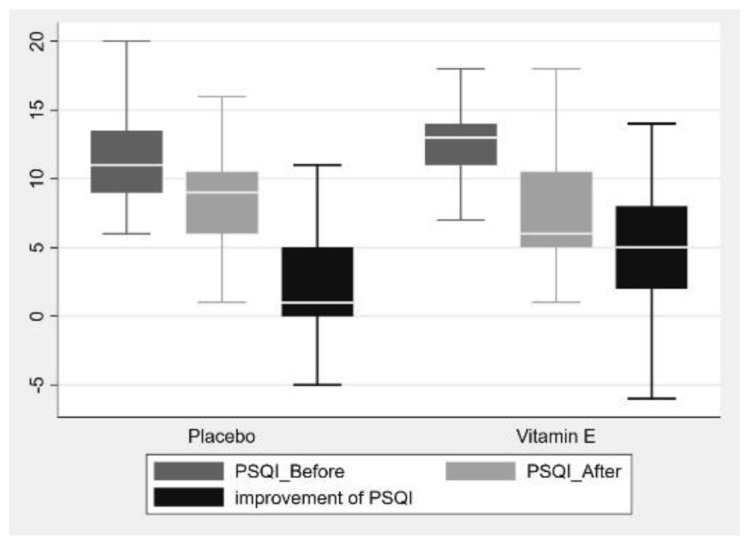
Boxplot of the PSQI score before and after intervention between the two groups.

**Figure 3:**
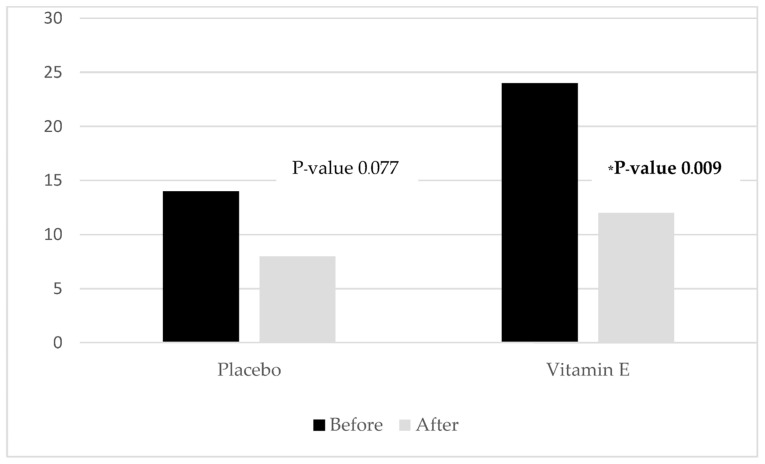
Histogram representing the reduction of sedative drug percentage used in each group. * statistical significance.

Furthermore, the improvement of sleep quality was significantly greater in the vitamin E group compared with the placebo group (5 (−6, 14) vs. 1 (−5, 13); *p*-value 0.001), as shown in Table 2 and Figure 2. With regard to the secondary outcome, the prevalence of sedative drug use in the vitamin E group decreased from 30% to 15% (*p*-value 0.004). In comparison, there was no statistically significant reduction in the placebo group from 17.5% to 10% (*p*-value 0.077), as shown in Table 3 and Figure 3.

## 4. Discussion

The main findings of our study indicate that a one-month vitamin E prescription can improve sleep quality and reduce sedative drug use in postmenopausal women with chronic insomnia disorder. There is much evidence to back up the benefits of vitamin E in menopause, whether it is for postmenopausal symptoms or bone health [27,28,29,30]. However, as described previously, no clinical trial has been conducted to evaluate the potential effect of vitamin E on chronic insomnia disorder, especially in postmenopausal women. Currently, apart from CBT-I, menopausal hormone therapy, or sedative drugs, two alternative agents (Valerian and isoflavones) can be used for treating chronic insomnia in menopausal women [12,39]. In 2011, Taavoni and colleagues conducted a triple-blinded, randomized controlled trial in a population comprising 100 postmenopausal women with self-reported insomnia. All the participants were randomized and divided equally into two groups. The first group took a pill containing 530 mg of valerian extract, taken once daily, versus a placebo. The investigators assessed sleep quality before and after four weeks using the Pittsburgh Sleep Quality Index questionnaire and found that the valerian extract supplementation group had a statistically significant reduction in PSQI score compared to the placebo group. Moreover, sleep quality increased by 30% in the valerian group compared to just 4% in the placebo group [40]. Hachul et al. conducted a double-blinded, randomized controlled trial in 2011. The study divided thirty-eight participants into two groups: women who were prescribed 80 mg/day of isoflavones, a naturally occurring substance found in soy, and those who were given a placebo. The authors compared the measured values from polysomnography after four months of intervention. They found that in the isoflavone group, the sleep efficiency from polysomnography showed a statistically significant improvement compared to the placebo group [41].

Despite these discoveries, the current knowledge about the relationship between vitamin E and sleep quality improvement is based only on descriptive data or studies in animal models. An exploratory study in 2008 by Lichstein et al. surveyed a total population of 519 men and women aged 20–98 in Shelby County, Tennessee. The study aimed to assess changes in sleep quality associated with vitamins. When separating the subgroups, the participants who were only prescribed vitamin E had better sleep quality and scores than those with no vitamin use [37]. However, the study had many limitations, such as the study design, the method for data collection, and the small number of participants who were only prescribed vitamin E. Furthermore, the population in the study had a wide range of demographic characteristics, whereas our study is more specific. The study with the most potential to evaluate the molecular mechanism for the effect of vitamin E on sleep deprivation was conducted in 2011 by Alzoubi and colleagues on mice divided into five cages, some of which were induced into a state of sleep deprivation. Vitamin E was fed to the chronically sleep-deprived mice for six weeks, and behavioral changes, spatial learning, and memory were assessed periodically. In addition, the oxidative stress markers were assessed by a calorimetric immunoassay method after the dissection of the hippocampus. In terms of oxidative stress, learning ability, and both short- and long-term memory, those prescribed vitamin E performed better, experienced less oxidative stress, and had better antioxidant level markers [42].

The PSQI questionnaire comprises seven domains: sleep quality, sleep latency, sleep duration, sleep efficiency, sleep disturbance, sleep medication use, and daytime dysfunction, in all of which the chronic stress stage is involved. Vitamin E supplementation may lower the stage of stress or resolve its chronicity through its ability to improve biomarkers of oxidative stress, described earlier in the proposed mechanism. The improvement score of the vitamin E group in this study was much greater than that of the placebo group, indicating that vitamin E improved sleep quality. When exploring micronutrient inadequacy and sleep in a large general population, Ikonte and colleagues discovered that women with adequate intakes of vitamin E below 60% were more likely to suffer from short sleep than the population as a whole [43]. Although the study was cross-sectional, its large sample size may justify using it to support the findings of our clinical trial. Our secondary objective was the reduction in sedative drugs, and we found the reduction in the percentage of women using them to be significantly greater in the vitamin E group versus the placebo. In Thailand, the most commonly used sedative drugs in outpatient clinics were in the benzodiazepine group (lorazepam, alprazolam, and diazepam) [44], which cause side effects such as drowsiness, sedated state, or dizziness. These side effects were disliked, especially in postmenopausal women, because they increased the risk of falling or decreased self-response to perturbations [45,46,47], which vitamin E could potentially reduce.

To our knowledge, the strength of this study is that it was the first randomized placebo-controlled trial to evaluate vitamin E’s effects on chronic insomnia disorder. Another strength is that our study chose the PSQI questionnaire, which is simple, internationally standardized, and validated in its Thai version for identifying poor sleepers and following up on sleep quality. The main limitation is that we did not evaluate serum vitamin E or oxidative stress markers before and after the intervention. Second, the PSQI questionnaire was designed only to evaluate sleep quality within one month, so the long-term result of a vitamin E prescription for chronic insomnia disorder is still unknown and requires further study. Third, the baseline PSQI scores in the vitamin E group were higher than the placebo group. Although we used a randomization design intending to have similar sleep quality between the two participant groups, this situation could happen by chance. While worse sleep quality in the intervention group was observed, there was more room for improvement which might raise some concern that the actual effect of vitamin E on relieving insomnia would be obscured. Nevertheless, the difference between the baseline PSQI scores of both participant groups was only two, which may not be clinically significant. Furthermore, the improvement score and rate of sedative drugs used were much better in the vitamin E group, reflecting some benefits of vitamin E supplements on sleep. Chronic insomnia disorder is a complex disease with multiple unidentified causes and various treatments available. Management options include non-pharmacological and pharmacological treatments, as mentioned in the introduction. At the same time, oxidative stress plays a role in chronic insomnia, which is improved by potent fat-soluble antioxidant supplementation, or vitamin E, as shown in our results. As a result, future research should correlate the oxidative stress level, the anti-oxidative level, the sleep disturbance, and changes in pre- and post-antioxidant, or vitamin E, supplements to further investigate this relationship.

## 5. Conclusions

Chronic insomnia disorder is one of the most common problems among postmenopausal women, with various treatment strategies available. We have examined vitamin E’s potential as an alternative treatment for chronic insomnia disorder that improves sleep quality and reduces sedative drug use.

## Figures and Tables

**Table 1:** Baseline characteristics of the participants.

Characteristics	Vitamin E (*N* = 80)	Placebo (*N* = 80)
Age (years) ^b^	57 (53, 59)	55 (52, 60)
Body mass index (kg/m^2^) ^b^	23.4 (20.7, 25.8)	22.9 (21.4, 25.9)
Age at menopause (years) ^a^	50.4 ± 3	49.7 ± 2.7
Years since menopause (years) ^a^	6.8 ± 5.4	7.1 ± 5.3
Type of menopause ^c^		
Natural	63 (78.75%)	69 (86.25%)
Surgical	17 (21.25%)	11 (13.75%)
Marital status ^c^		
Single	12 (15%)	15 (18.75%)
Married	51 (63.75%)	55 (68.75%)
Divorced or widowed	17 (21.25%)	10 (12.5%)
Number of children ^c^		
0	17 (21.25%)	20 (25%)
1	25 (31.25%)	18 (22.5%)
≥2	38 (47.5%)	42 (52.5%)
Education ^c^		
Primary school	4 (5%)	9 (11.25%)
High school	23 (28.75%)	23 (28.75%)
Bachelor’s degree or higher	53 (66.25%)	48 (60%)
Underlying diseases ^c^		
Yes	35 (43.75%)	38 (47.5%)
No	45 (56.25%)	42 (52.5%)
Sedative drug used ^c^	24 (30%)	14 (17.5%)
Caffeine consumption ^c^		
<3 drinks per week	6 (7.5%)	11 (13.75%)
>3 drinks per week	19 (23.75%)	17 (21.25%)
Financial status ^c^		
Bad	3 (3.75%)	6 (7.5%)
Fair	61 (76.25%)	62 (77.5%)
Good	16 (20%)	12 (15%)

Notes: ^a^ Data expressed as mean ± standard deviation (SD). ^b^ Data expressed as median (range). ^c^ Data expressed as numbers (percentage).

**Table 2:** The Pittsburgh Sleep Quality Index comparison between the two groups.

PSQI Score	Vitamin E (*N* = 80)	Placebo (*N* = 80)	*p*-Value
Before	13 (6, 20)	11 (6, 20)	**0.019**
After	6 (1, 18)	9 (1, 19)	0.012
Improvement of PSQI	5 (−6, 14)	1 (−5, 13)	<0.001

Notes: Data expressed as median (range).

**Table 3:** Sedative drug use in each group.

Sedative Drug Used	Vitamin E	Placebo
Before	24 (30%)	14 (17.5%)
After	12 (15%)	8 (10%)
*p*-Value	0.009	0.077

## Data Availability

The data are available on request due to restrictions on privacy or ethics. The data presented in this study are available on request from the corresponding author. The data are not publicly available due to the ethics and the right of the faculty of medicine, Ramathibodi Hospital, Mahidol University.

## References

[1] Gold E.B. (2011). The Timing of the Age at Which Natural Menopause Occurs. Obstet. Gynecol. Clin. N. Am..

[2] Institute for Population and Social Research, Mahidol UniversityMahidol Population Gazette. https://pr.mahidol.ac.th/ipsrbeta/en/Gazette.aspx.

[3] Santoro N., Epperson C.N., Mathews S.B. (2015). Menopausal Symptoms and Their Management. Endocrinol. Metab. Clin. N. Am..

[4] Riemann D., Baglioni C., Bassetti C., Bjorvatn B., Groselj L.D., Ellis J.G., Espie C.A., Garcia-Borreguero D., Gjerstad M., Gonçalves M. (2017). European guideline for the diagnosis and treatment of insomnia. J. Sleep Res..

[5] Patel D., Steinberg J., Patel P. (2018). Insomnia in the Elderly: A Review. J. Clin. Sleep Med..

[6] Ohayon M.M. (2002). Epidemiology of insomnia: What we know and what we still need to learn. Sleep Med. Rev..

[7] Joffe H., Massler A., Sharkey K. (2010). Evaluation and Management of Sleep Disturbance during the Menopause Transition. Semin. Reprod. Med..

[8] Pengo M., Won C.H., Bourjeily G. (2018). Sleep in Women Across the Life Span. Chest.

[9] Young T., Rabago D., Zgierska A., Austin D., Finn L. (2003). Objective and Subjective Sleep Quality in Premenopausal, Perimenopausal, and Postmenopausal Women in the Wisconsin Sleep Cohort Study. Sleep.

[10] Xu M., Bélanger L., Ivers H., Guay B., Zhang J., Morin C.M. (2011). Comparison of subjective and objective sleep quality in menopausal and non-menopausal women with insomnia. Sleep Med..

[11] Ge L., Guyatt G., Tian J., Pan B., Chang Y., Chen Y., Li H., Zhang J., Li Y., Ling J. (2019). Insomnia and risk of mortality from all-cause, cardiovascular disease, and cancer: Systematic review and meta-analysis of prospective cohort studies. Sleep Med. Rev..

[12] Attarian H., Hachul H., Guttuso T., Phillips B. (2015). Treatment of chronic insomnia disorder in menopause: Evaluation of literature. Menopause.

[13] Parthasarathy S., Vasquez M.M., Halonen M., Bootzin R., Quan S.F., Martinez F.D., Guerra S. (2014). Persistent Insomnia is Associated with Mortality Risk. Am. J. Med..

[14] Sivertsen B., Pallesen S., Glozier N., Bjorvatn B., Salo P., Tell G.S., Ursin R., Øverland S. (2014). Midlife insomnia and subsequent mortality: The Hordaland health study. BMC Public Health.

[15] Kay-Stacey M., Attarian H. (2016). Advances in the management of chronic insomnia. BMJ.

[16] Ciano C., King T.S., Wright R.R., Perlis M., Sawyer A.M. (2017). Longitudinal Study of Insomnia Symptoms Among Women During Perimenopause. J. Obstet. Gynecol. Neonatal Nurs..

[17] Punyahotra S., Dennerstein L., Lehert P. (1997). Menopausal experiences of Thai women. Part 1: Symptoms and their correlates. Maturitas.

[18] Yazdi Z., Sadeghniiat-Haghighi K., Ziaee A., Elmizadeh K., Ziaeeha M. (2013). Influence of Sleep Disturbances on Quality of Life of Iranian Menopausal Women. Psychiatry J..

[19] Lloret A., Esteve D., Lloret M.A., Monllor P., López B., León J.L., Cervera-Ferri A. (2021). Is Oxidative Stress the Link Between Cerebral Small Vessel Disease, Sleep Disruption, and Oligodendrocyte Dysfunction in the Onset of Alzheimer’s Disease?. Front. Physiol..

[20] Atrooz F., Salim S. (2020). Sleep deprivation, oxidative stress and inflammation. Adv. Protein. Chem. Struct. Biol..

[21] Islam M.T. (2017). Oxidative stress and mitochondrial dysfunction-linked neurodegenerative disorders. Neurol. Res..

[22] El-Helaly M., Abu-Hashem E. (2010). Oxidative stress, melatonin level, and sleep insufficiency among electronic equipment repairers. Indian J. Occup. Environ. Med..

[23] Gulec M., Ozkol H., Selvi Y., Tuluce Y., Aydin A., Besiroglu L., Ozdemir P.G. (2012). Oxidative stress in patients with primary insomnia. Prog. Neuro-Psychopharmacology Biol. Psychiatry.

[24] Feng L., Wu H.-W., Song G.-Q., Lu C., Li Y.-H., Qu L.-N., Chen S.-G., Liu X.-M., Chang Q. (2016). Chronical sleep interruption-induced cognitive decline assessed by a metabolomics method. Behav. Brain Res..

[25] Azzi A. (2019). Tocopherols, tocotrienols and tocomonoenols: Many similar molecules but only one vitamin E. Redox Biol..

[26] Institute of Medicine (US) Panel on Dietary Antioxidants and Related Compounds (2000). Dietary Reference Intakes for Vitamin C, Vitamin E, Selenium, and Carotenoids.

[27] Ziaei S., Kazemnejad A., Zareai M. (2007). The Effect of Vitamin E on Hot Flashes in Menopausal Women. Gynecol. Obstet. Investig..

[28] Vallibhakara S.A.-O., Nakpalat K., Sophonsritsuk A., Tantitham C., Vallibhakara O. (2021). Effect of Vitamin E Supplement on Bone Turnover Markers in Postmenopausal Osteopenic Women: A Double-Blind, Randomized, Placebo-Controlled Trial. Nutrients.

[29] Guralp O. (2014). Effects of vitamin E on bone remodeling in perimenopausal women: Mini review. Maturitas.

[30] Ochi H., Takeda S. (2014). The Two Sides of Vitamin E Supplementation. Gerontology.

[31] Abner E.L., Schmitt F.A., Mendiondo M.S., Marcum J., Kryscio R.J. (2011). Vitamin E and All-Cause Mortality: A Meta-Analysis. Curr. Aging Sci..

[32] Mustapha M., Nassir C.M.N.C.M., Hay Y.K., Yee F.W., Hamid H.A., Otero-Losada M., Capani F., Lloret S.P. (2020). Neuroprotective Potentials of Natural Vitamin E for Cerebral Small Vessel Disease. Neuroprotection—New Approaches and Prospects.

[33] Jain S.K., McVie R., Smith T. (2000). Vitamin E supplementation restores glutathione and malondialdehyde to normal concentrations in erythrocytes of type 1 diabetic children. Diabetes Care.

[34] Bergin P., Leggett A., Cardwell C.R., Woodside J.V., Thakkinstian A., Maxwell A.P., McKay G.J. (2021). The effects of vitamin E supplementation on malondialdehyde as a biomarker of oxidative stress in haemodialysis patients: A systematic review and meta-analysis. BMC Nephrol..

[35] Buysse D.J., Reynolds CF 3rd Monk T.H., Berman S.R., Kupfer D.J. (1989). The Pittsburgh Sleep Quality Index: A new instrument for psychiatric practice and research. Psychiatry Res..

[36] Sitasuwan T., Bussaratid S., Ruttanaumpawan P., Chotinaiwattarakul W. (2014). Reliability and validity of the Thai version of the Pittsburgh Sleep Quality Index. J. Med. Assoc. Thail..

[37] Lichstein K.L., Payne K.L., Soeffing J.P., Durrence H.H., Taylor D.J., Riedel B.W., Bush A.J. (2007). Vitamins and sleep: An exploratory study. Sleep Med..

[38] Ngamjarus C. (2016). n4Studies: Sample Size Calculation for an Epidemiological Study on a Smart Device. Siriraj Med. J..

[39] Proserpio P., Marra S., Campana C., Agostoni E.C., Palagini L., Nobili L., Nappi R.E. (2020). Insomnia and menopause: A narrative review on mechanisms and treatments. Climacteric.

[40] Taavoni S., Ekbatani N., Kashaniyan M., Haghani H. (2011). Effect of valerian on sleep quality in postmenopausal women: A randomized placebo-controlled clinical trial. Menopause.

[41] Hachul H., Brandão L.C., D’Almeida V., Bittencourt L., Baracat E.C., Tufik S. (2011). Isoflavones decrease insomnia in postmenopause. Menopause.

[42] Alzoubi K.H., Khabour O.F., Rashid B.A., Damaj I.M., Salah H.A. (2012). The neuroprotective effect of vitamin E on chronic sleep deprivation-induced memory impairment: The role of oxidative stress. Behav Brain Res..

[43] Ikonte C.J., Mun J.G., Reider C.A., Grant R.W., Mitmesser S.H. (2019). Micronutrient Inadequacy in Short Sleep: Analysis of the NHANES 2005–2016. Nutrients.

[44] Prueksaritanond S., Tubtimtes S., Pumkompol T., Sukying C. (2009). Psychotropic drug prescribing in the family medicine out-patient clinic, Ramathibodi Hospital. J. Med Assoc. Thail..

[45] Herzig S.J., Rothberg M.B., Moss C.R., Maddaleni G., Bertisch S.M., Wong J., Zhou W., Ngo L., Anderson T.S., Gurwitz J.H. (2021). Risk of in-hospital falls among medications commonly used for insomnia in hospitalized patients. Sleep.

[46] Aljawadi M.H., Khoja A.T., Alhammad A.M., AlOtaibi A.D., Al-Shammari S.A., Khoja T.A. (2018). The prevalence of benzodiazepines utilization and its association with falls among Saudi older adults; results from the Saudi national survey for elderly Health (SNSEH). Saudi Pharm. J..

[47] Richardson J.K., Eckner J.T., Kim H., Ashton-Miller J.A. (2020). A clinical method of evaluating simple reaction time and reaction accuracy is sensitive to a single dose of lorazepam. J. Psychopharmacol..

